# Human Leukocyte Antigen and Systemic Sclerosis in Japanese: The Sign of the Four Independent Protective Alleles, DRB1*13:*02*, DRB1*14:*06*, DQB1*03:*01*, and DPB1*02:*01*

**DOI:** 10.1371/journal.pone.0154255

**Published:** 2016-04-26

**Authors:** Hiroshi Furukawa, Shomi Oka, Aya Kawasaki, Kota Shimada, Shoji Sugii, Takashi Matsushita, Atsushi Hashimoto, Akiko Komiya, Naoshi Fukui, Kouji Kobayashi, Atsumu Osada, Atsushi Ihata, Yuya Kondo, Tatsuo Nagai, Keigo Setoguchi, Akiko Okamoto, Akira Okamoto, Noriyuki Chiba, Eiichi Suematsu, Hajime Kono, Masao Katayama, Shunsei Hirohata, Takayuki Sumida, Kiyoshi Migita, Minoru Hasegawa, Manabu Fujimoto, Shinichi Sato, Shouhei Nagaoka, Kazuhiko Takehara, Shigeto Tohma, Naoyuki Tsuchiya

**Affiliations:** 1 Molecular and Genetic Epidemiology Laboratory, Faculty of Medicine, University of Tsukuba, 1-1-1 Tennodai, Tsukuba, Japan; 2 Clinical Research Center for Allergy and Rheumatology, Sagamihara Hospital, National Hospital Organization, 18-1 Sakuradai, Minami-ku, Sagamihara, Japan; 3 Department of Rheumatology, Sagamihara Hospital, National Hospital Organization, 18-1 Sakuradai, Minami-ku, Sagamihara, Japan; 4 Department of Rheumatic Diseases, Tokyo Metropolitan Tama Medical Center, 2-8-29 Musashi-dai, Fuchu, Japan; 5 Department of Dermatology, Faculty of Medicine, Institute of Medical, Pharmaceutical, and Health Sciences, Kanazawa University, 13-1 Takaramachi, Kanazawa, Japan; 6 Department of Rheumatology, Yokohama Minami Kyosai Hospital, 1-21-1 Rokuura-higashi, Kanazawa-ku, Yokohama, Japan; 7 Department of Internal Medicine, Faculty of Medicine, University of Tsukuba, 1-1-1 Tennodai, Tsukuba, Japan; 8 Department of Rheumatology and Infectious Diseases, Kitasato University School of Medicine, 1-15-1 Kitasato, Minami-ku, Sagamihara, Japan; 9 Department of Allergy and Immunological Diseases, Tokyo Metropolitan Cancer and Infectious Diseases Center Komagome Hospital, 3-18-22 Hon-komagome, Bunkyo-ku, Tokyo, Japan; 10 Department of Internal Medicine, Teikyo University, 2-11-1 Kaga, Itabashi-ku, Tokyo, Japan; 11 Department of Rheumatology, Himeji Medical Center, National Hospital Organization, 68 Hon-machi, Himeji, Japan; 12 Department of Rheumatology, Morioka Hospital, National Hospital Organization, 1-25-1 Aoyama, Morioka, Japan; 13 Department of Internal Medicine and Rheumatology, Clinical Research Institute, Kyushu Medical Center, National Hospital Organization, 1-8-1 Jigyohama, Chuo-ku, Fukuoka, Japan; 14 Department of Internal Medicine, Nagoya Medical Center, National Hospital Organization, 4-1-1 Sannomaru, Naka-ku, Nagoya, Japan; 15 Clinical Research Center, Nagasaki Medical Center, National Hospital Organization, 2-1001-1 Kubara, Omura, Japan; 16 Department of Dermatology, School of Medicine, Faculty of Medical Sciences, University of Fukui, 23-3 Matsuokashimoaizuki, Eiheiji, Japan; 17 Department of Dermatology, Faculty of Medicine, University of Tsukuba, 1-1-1 Tennodai, Tsukuba, Japan; 18 Department of Dermatology, Graduate School of Medicine, University of Tokyo, 7-3-1 Hongo, Bunkyo-ku, Tokyo, Japan; University of Texas Health Science Center at Houston, UNITED STATES

## Abstract

**Objective:**

Several studies on associations between human leukocyte antigen (HLA) allele frequencies and susceptibility to systemic sclerosis (SSc) have been reported. Anti-centromere antibodies (ACA) and anti-topoisomerase I antibodies (ATA) are found in SSc patients. Here, we sought to identify *HLA* alleles associated with SSc in Japanese, and explored their associations with SSc phenotypes including the presence of autoantibodies.

**Methods:**

Associations of *HLA-DRB1*, *DQB1*, *and DPB1* were analyzed in 463 Japanese SSc patients and 413 controls.

**Results:**

We found that DRB1*13:*02* (*P* = 0.0011, *P*c = 0.0319, odds ratio [OR] 0.46, 95% confidence interval [CI] 0.29–0.73), DRB1*14:*06* (*P* = 6.60X10^-5^, *Pc* = 0.0020, OR 0.05, 95%CI 0.01–0.41), DQB1*03:*01* (*P* = 0.0009, *Pc* = 0.0150, OR 0.56, 95%CI 0.40–0.79), and DPB1*02:*01* (*P* = 5.16X10^-6^, *Pc* = 8.77X10^-5^, OR 0.52, 95%CI 0.39–0.69) were protectively associated with SSc. In addition, these four alleles seemed to be independently associated with the protection against the susceptibility of SSc. On the other hand, we could not find predisposing alleles for overall SSc. With respect to SSc subsets, a tendency for these four alleles to be protectively associated was observed. However, there was a significant association between DRB1*01:*01*, DRB1*10:*01*, DQB1*05:*01*, and DPB1*04:*02* and the susceptibility to SSc with ACA. On the other hand, the presence of DRB1*15:*02*, DQB1*06:*01*, DPB1*03:*01*, and DPB1*09:*01* was associated with SSc with ATA.

**Conclusion:**

Thus, the present study has identified protective associations of the four *HLA* class II alleles with overall Japanese SSc and predisposing associations of *HLA* class II alleles with Japanese SSc subsets.

## Introduction

Systemic sclerosis (SSc) is a complex autoimmune disease of unknown etiology that is characterized by fibrosis of the skin and internal organs, small-vessel vasculopathy, and the production of anti-nuclear antibodies. It is a chronic autoimmune disease, susceptibility to which is associated with genetic and environmental factors [1,2]. Genetic risk factors for SSc include alleles of the loci *HLA-DRB1*, *DQB1*, *DPB1*, *DPB2*, *IRF5*, *STAT4*, *CD247*, *IRF4*, and others [1,3,4,5]. Thus, a functional role of these polymorphisms in SSc has been suggested, as well as relationships with other autoimmune diseases such as rheumatoid arthritis or systemic lupus erythematosus. In particular, skewed frequencies of human leukocyte antigen (*HLA*) alleles are known to be associated with SSc. Different *HLA* class II alleles appear to be associated with SSc susceptibility in different ethnic groups, such as HLA-DRB1*11:*04*, DQB1*03:*01*, or DQB1*26 epi (*DQB1* alleles encoding a non-leucine residue at position 26) in Europeans[2,6,7], DRB1*08:*04*, DQB1*03:*01*, or DPB1*13:*01* in African-Americans [6], and DRB1*15:*02*, DQB1*05:*01*, and DPB1*03:*01* in Asians [8,9,10,11,12,13,14].

SSc presents with several specific autoantibodies including anti-centromere antibodies (ACA) [15] and anti-topoisomerase I antibodies (ATA, also termed Scl-70) [16]. ACA are present in a subset of patients with SSc who have limited cutaneous SSc (lcSSc). This is characterized by skin thickening that is relatively restricted to the fingers and hands, with less severe internal organ involvement. ATA are present in an SSc subset having diffuse cutaneous SSc (dcSSc), in which skin lesions are extensive and progressive, and serious internal organ involvement is observed. DQB1*05:*01*, DQB1*26 epi are associated with SSc with ACA in people of European descent [6] and DQB1*05:*01* in Japanese [9]. Several studies have also shown that certain *DRB1* or *DPB1* alleles are associated with SSc with ATA; thus, DPB1*13:*01* is associated with SSc with ATA in Europeans [6] and DRB1*15:*02* and DPB1*09:*01* in Japanese [9].

Here, we sought *HLA* alleles predisposing and protective to SSc in Japanese, and explored their associations with SSc phenotypes including the presence of autoantibodies.

## Materials and Methods

### Patients and healthy controls

SSc patients were recruited at Sagamihara Hospital, Yokohama Minami Kyosai Hospital, Tama Medical Center, Kitasato University, Komagome Hospital, Teikyo University, Himeji Medical Center, Morioka Hospital, Kyushu Medical Center, Nagoya Medical Center, Nagasaki Medical Center, University of Tsukuba, and Kanazawa University. Healthy controls (n = 413; mean age ± SD, 41.4 ± 12.6 years, 62 male [14.0%]) were recruited without matching at Sagamihara Hospital, Teikyo University, and Kanazawa University or by the Pharma SNP Consortium (Tokyo, Japan) [17]. All patients and healthy individuals were native Japanese living in Japan. All patients with SSc fulfilled the American College of Rheumatology criteria for SSc [18] and were classified as dcSSc and lcSSc according to the classification criteria by LeRoy et al. [19]. ACA was detected using Mesacup-2 test CENP-B enzyme-linked immunosorbent assay (ELISA, Medical & Biological Laboratories, Nagoya, Japan). ATA was detected using Mesacup-3 test Scl-70 ELISA (Medical & Biological Laboratories) or Ouchterlony double immunodiffusion method (TFB, Hachioji, Japan). This study was reviewed and approved by the research ethics committees of each participating institute as follows: Sagamihara Hospital Research Ethics Committee, Yokohama Minami Kyosai Hospital Research Ethics Committee, Tama Medical Center Research Ethics Committee, Kitasato University Research Ethics Committee, Komagome Hospital Research Ethics Committee, Teikyo University Research Ethics Committee, Himeji Medical Center Research Ethics Committee, Morioka Hospital Research Ethics Committee, Kyushu Medical Center Research Ethics Committee, Nagoya Medical Center Research Ethics Committee, Nagasaki Medical Center Research Ethics Committee, Kanazawa University Research Ethics Committee, and University of Tsukuba Research Ethics Committee. Written informed consent was obtained from all study participants. This study was conducted in accordance with the principles expressed in the Declaration of Helsinki.

### Genotyping

Genotyping of *HLA-DRB1*, *DQB1*, and *DPB1* was performed by the polymerase chain reaction technique with sequence-specific oligonucleotide probes (WAKFlow HLA typing kits, Wakunaga, Hiroshima, Japan), using the Bio-Plex 200 system (Bio-Rad, Hercules, CA). DQB1*26 epi alleles are DQB1*03:*01*, *04:*01*, *04:*02*, *05:*01*, *05:*02*, *05:*03*, and *06:*01*. Results of genotyping for some of the healthy controls were reported previously [20,21,22].

### Statistical analysis

Differences of SSc characteristics were analyzed by Student's t-test or Fisher’s exact test using 2X2 contingency tables. The Hardy-Weinberg exact tests were performed by the Markov chain method under the condition of 10000 each of dememorization, batches, and iterations per batch (Genepop on the web; http://genepop.curtin.edu.au/) [23]. The statistical power in each condition of allele carrier frequency and odds ratio was calculated on the sample size of this study (463 overall SSc patients and 413 controls, S1 Fig) by PS: Power and Sample Size Calculation version 3.1.2 (http://biostat.mc.vanderbilt.edu/wiki/Main/PowerSampleSize) [24]. Differences of allele carrier frequencies were analyzed by Fisher’s exact test using 2X2 contingency tables under the dominant model. Adjustment for multiple comparisons was performed using the Bonferroni method. Corrected *P* (*P*c) values were calculated by multiplying the *P* value by the number of alleles tested. Relative predispositional effects (RPE) were analyzed by sequential elimination of carriers of each allele with the strongest association [25]. To examine whether each protective *HLA* class II allele independently contributes to the protection of SSc, multiple logistic regression analysis under the additive model was employed and the deviation from 0 was evaluated for coefficients by the Wald test. *DRB1-DQB1-DPB1* haplotype frequencies were estimated using the expectation-maximization method by SNPAlyze ver.8.0.4 Pro software (Dynacom, Chiba, Japan). *P* values were calculated by permutation test (100000 permutations). Differences of amino acid residue carrier frequencies (amino acid residue carrier vs. non-carrier) were analyzed by Fisher’s exact test using 2X2 contingency tables under the dominant model on the detected polymorphic amino acid positions in the β1 domain of HLA-DRβ, DQβ, and DPβ chains. Adjustment for multiple comparisons was performed using the Bonferroni method. *P*c values were calculated by multiplying the *P* value by the number of amino acid positions tested.

## Results

### Clinical features of the SSc patients

Characteristics of the SSc patients are described in Table 1. Mean age and ACA positivity in dcSSc were lower than in lcSSc. ATA positivity and percentage of male were higher in dcSSc than lcSSc.

**Table 1:** Characteristics of the SSc patients.

	SSc	dcSSc	lcSSc	*P*
Number	463	157	266	
Mean age, years (SD)	58.4 (13.5)	54.5 (14.9)	61.0 (12.0)	*4.65X10^-6^
Male, n (%)	50 (10.9)	29 (18.5)	19 (7.2)	0.0007
ACA positive, n (%)	194 (44.9)	20 (12.9)	167 (65.0)	1.73X10^-26^
ATA positive, n (%)	119 (27.4)	85 (54.5)	27 (10.5)	3.17X10^-22^

SSc: systemic sclerosis, dcSSc: diffuse cutaneous SSc, lcSSc: limited cutaneous SSc, ACA: anti-centromere antibodies, ATA: anti-topoisomerase I antibodies. Association was tested between dcSSc and lcSSc by Fisher's exact test using 2X2 contingency tables or Student's t-test.

* Student's t-test was employed.

### *HLA* association analysis of SSc patients

*HLA-DRB1*, *DQB1*, *and DPB1* genotyping was performed in SSc patients and healthy controls to compare carrier frequencies of each allele. No deviation from Hardy-Weinberg equilibrium was detected in the controls (*DRB1*: *P* = 0.4327, *DQB1*: *P* = 0.2136, *DPB1*: *P* = 0.7464, all locus: *P* = 0.5000), though a deviation was observed in the overall SSc patients (*DRB1*: *P* = 0.1017, *DQB1*: *P* = 0.0769, *DPB1*: *P* = 0.0260, all locus: *P* = 0.0093). A significant protective association was found for DRB1*13:*02* (*P* = 0.0011, *P*c = 0.0319, odds ratio [OR] 0.46, 95% confidence interval [CI] 0.29–0.73, Table 2) and DRB1*14:*06* (*P* = 6.60X10^-5^, *P*c = 0.0020, OR 0.05, 95%CI 0.01–0.41, Table 2) with SSc. A significant association with resistance to SSc was also found for the DR6 serological group (DRB1*13 and DRB1*14, *P* = 7.08X10^-6^, OR 0.49, 95%CI 0.36–0.67, Table 2). We further explored associations between *DRB1* alleles and SSc using RPE testing by sequential elimination of carriers of each allele with the strongest association (Table 2, right column). The prime strongest association was between SSc and DRB1*14:*06*, followed by *13:*02*, *10:*01*, *04:*03*, and *04:*07*. A protective association between the carrier frequency of DQB1*03:*01* (*P* = 0.0009, *P*c = 0.0150, OR 0.56, 95% CI 0.40–0.79, Table 3) or DPB1*02:*01* (*P* = 5.16X10^-6^, *P*c = 8.77X10^-5^, OR 0.52, 95% CI 0.39–0.69, Table 3) and SSc was detected. The associations between *DQB1* or *DPB1* alleles and SSc using RPE were also analyzed. RPE were tested by sequential elimination of carriers of each of the *DQB1* alleles DQB1*03:*01*, *06:*04*, and *06:*02*; *DPB1* alleles DPB1*02:*01* and *03:*01*, respectively. No statistically significant predisposing associations were found for DQB1*26 epi (*P* = 0.2443, OR 1.24) or any HLA class II alleles. Thus, lower carrier frequencies of the four class II alleles, DRB1*13:*02*, DRB1*14:*06*, DQB1*03:*01*, and DPB1*02:*01*, were present in SSc patients.

**Table 2:** *HLA-DRB1* allele carrier frequencies in the SSc patients and the healthy controls.

	Case (n = 463)	Control (n = 413)	*P*	OR	*P*c	95%CI	*P* (RPE)
DRB1*01:*01*	65 (14.0)	42 (10.2)	0.0979	1.44	NS		
DRB1*03:*01*	1 (0.2)	2 (0.5)	0.6044	0.44	NS		
DRB1*04:*01*	10 (2.2)	7 (1.7)	0.8071	1.28	NS		
DRB1*04:*03*	39 (8.4)	19 (4.6)	0.0288	1.91	0.8633	(1.08–3.36)	0.0343
DRB1*04:*04*	6 (1.3)	0 (0.0)	0.0322	11.75	0.9672	(0.66–209.22)	
DRB1*04:*05*	94 (20.3)	87 (21.1)	0.8023	0.95	NS		
DRB1*04:*06*	31 (6.7)	34 (8.2)	0.4390	0.80	NS		
DRB1*04:*07*	11 (2.4)	3 (0.7)	0.0610	3.33	NS		0.0439
DRB1*04:*10*	23 (5.0)	14 (3.4)	0.3129	1.49	NS		
DRB1*07:*01*	7 (1.5)	3 (0.7)	0.3490	2.10	NS		
DRB1*08:*02*	56 (12.1)	38 (9.2)	0.1896	1.36	NS		
DRB1*08:*03*	79 (17.1)	61 (14.8)	0.4059	1.19	NS		
DRB1*08:*09*	0 (0.0)	1 (0.2)	0.4715	0.30	NS		
DRB1*09:*01*	116 (25.1)	105 (25.4)	0.9379	0.98	NS		
DRB1*10:*01*	15 (3.2)	2 (0.5)	0.0027	6.88	0.0820	(1.56–30.27)	0.0059
DRB1*11:*01*	17 (3.7)	22 (5.3)	0.2540	0.68	NS		
DRB1*12:*01*	26 (5.6)	29 (7.0)	0.4058	0.79	NS		
DRB1*12:*02*	7 (1.5)	10 (2.4)	0.3407	0.62	NS		
DRB1*13:*01*	4 (0.9)	5 (1.2)	0.7419	0.71	NS		
DRB1*13:*02*	32 (6.9)	57 (13.8)	0.0011	0.46	0.0319	(0.29–0.73)	0.0007
DRB1*14:*03*	15 (3.2)	21 (5.1)	0.1772	0.63	NS		
DRB1*14:*04*	0 (0.0)	1 (0.2)	0.4715	0.30	NS		
DRB1*14:*05*	21 (4.5)	14 (3.4)	0.4900	1.35	NS		
DRB1*14:*06*	1 (0.2)	16 (3.9)	6.60X10^-5^	0.05	0.0020	(0.01–0.41)	6.60X10^-5^
DRB1*14:*07*	0 (0.0)	1 (0.2)	0.4715	0.30	NS		
DRB1*14:*29*	1 (0.2)	0 (0.0)	1.0000	2.68	NS		
DRB1*14:*54*	23 (5.0)	28 (6.8)	0.3117	0.72	NS		
DRB1*15:*01*	55 (11.9)	68 (16.5)	0.0521	0.68	NS		
DRB1*15:*02*	120 (25.9)	89 (21.5)	0.1323	1.27	NS		
DRB1*16:*02*	5 (1.1)	5 (1.2)	1.0000	0.89	NS		
DR6	91 (19.7)	137 (33.2)	7.08X10^-6^	0.49		(0.36–0.67)	

SSc: systemic sclerosis, OR: odds ratio, CI: confidence interval, *P*c: corrected *P* value, NS: not significant, RPE: relative predispositional effects. Allele carrier frequencies are shown in parenthesis (%). Association was tested by Fisher's exact test using 2X2 contingency tables under the dominant model. RPE were tested by sequential elimination of carriers of each of the alleles DRB1*14:*06*, *13:*02*, *10:*01*, *04:*03*, and *04:*07*.

**Table 3:** *HLA-DQB1* and *DPB1* allele carrier frequencies in the SSc patients and the healthy controls.

	Case (n = 463)	Control (n = 413)	*P*	OR	*P*c	95%CI	*P* (RPE)
DQB1*02:*01*	1 (0.2)	2 (0.5)	0.6044	0.44	NS		
DQB1*02:*02*	7 (1.5)	3 (0.7)	0.3490	2.10	NS		
DQB1*03:*01*	67 (14.5)	96 (23.2)	0.0009	0.56	0.0150	(0.40–0.79)	0.0009
DQB1*03:*02*	112 (24.2)	83 (20.1)	0.1666	1.27	NS		
DQB1*03:*03*	124 (26.8)	111 (26.9)	1.0000	1.00	NS		
DQB1*03:*06*	1 (0.2)	0 (0.0)	1.0000	2.68	NS		
DQB1*04:*01*	91 (19.7)	86 (20.8)	0.6743	0.93	NS		
DQB1*04:*02*	51 (11.0)	29 (7.0)	0.0457	1.64	0.7312	(1.02–2.64)	
DQB1*05:*01*	79 (17.1)	44 (10.7)	0.0064	1.73	0.1030	(1.16–2.56)	
DQB1*05:*02*	16 (3.5)	16 (3.9)	0.8572	0.89	NS		
DQB1*05:*03*	31 (6.7)	34 (8.2)	0.4390	0.80	NS		
DQB1*06:*01*	185 (40.0)	144 (34.9)	0.1247	1.24	NS		
DQB1*06:*02*	54 (11.7)	65 (15.7)	0.0929	0.71	NS		0.0485
DQB1*06:*03*	4 (0.9)	6 (1.5)	0.5293	0.59	NS		
DQB1*06:*04*	30 (6.5)	50 (12.1)	0.0046	0.50	0.0741	(0.31–0.81)	0.0010
DQB1*06:*09*	3 (0.6)	6 (1.5)	0.3198	0.44	NS		
DPB1*02:*01*	128 (27.6)	175 (42.4)	5.16X10^-6^	0.52	8.77X10^-5^	(0.39–0.69)	5.16X10^-6^
DPB1*02:*02*	39 (8.4)	33 (8.0)	0.9020	1.06	NS		
DPB1*03:*01*	65 (14.0)	35 (8.5)	0.0105	1.76	0.1793	(1.14–2.72)	0.0315
DPB1*04:*01*	31 (6.7)	41 (9.9)	0.0858	0.65	NS		
DPB1*04:*02*	92 (19.9)	65 (15.7)	0.1133	1.33	NS		
DPB1*05:*01*	272 (58.7)	250 (60.5)	0.6293	0.93	NS		
DPB1*06:*01*	9 (1.9)	3 (0.7)	0.1511	2.71	NS		
DPB1*09:*01*	124 (26.8)	82 (19.9)	0.0167	1.48	0.2844	(1.07–2.03)	
DPB1*13:*01*	28 (6.0)	20 (4.8)	0.4605	1.26	NS		
DPB1*14:*01*	11 (2.4)	8 (1.9)	0.8171	1.23	NS		
DPB1*17:*01*	4 (0.9)	3 (0.7)	1.0000	1.19	NS		
DPB1*19:*01*	5 (1.1)	2 (0.5)	0.4566	2.24	NS		
DPB1*25:*01*	0 (0.0)	1 (0.2)	0.4715	0.30	NS		
DPB1*38:*01*	2 (0.4)	0 (0.0)	0.5011	4.48	NS		
DPB1*41:*01*	3 (0.6)	2 (0.5)	1.0000	1.34	NS		
DPB1*47:*01*	1 (0.2)	0 (0.0)	1.0000	2.68	NS		
DPB1*113:*01*	0 (0.0)	1 (0.2)	0.4715	0.30	NS		

SSc: systemic sclerosis, OR: odds ratio, CI: confidence interval, *P*c: corrected *P* value, NS: not significant, RPE: relative predispositional effects. Allele carrier frequencies are shown in parenthesis (%). Association was tested by Fisher's exact test using 2X2 contingency tables under the dominant model. RPE were tested by sequential elimination of carriers of each of the *DQB1* alleles DQB1*03:*01*, *06:*04*, and *06:*02; DPB1* alleles DPB1*02:*01* and *03:*01*, respectively.

*DRB1*, *DQB1*, and *DPB1* alleles are in linkage disequilibrium. In order to elucidate which of the four protective alleles was responsible for the observed protective associations, conditional logistic regression analysis between them in SSc was performed (Table 4). The association of DRB1*13:*02* remained significant, when conditioned on DRB1*14:*06*, DQB1*03:*01*, or DPB1*02:*01*. Similarly, the association of DRB1*14:*06* still remained significant, when conditioned on DRB1*13:*02*, DQB1*03:*01*, or DPB1*02:*01*. The significant association of DQB1*03:*01* was observed, when conditioned on DRB1*13:*02*, DRB1*14:*06*, or DPB1*02:*01*. The significant association of DPB1*02:*01* was also detected, when conditioned on DRB1*13:*02*, DRB1*14:*06*, or DQB1*03:*01*. Thus, significant protective associations for the four alleles with SSc were observed, when conditioned on each other, indicating an independent role for each protective allele in SSc.

**Table 4:** Conditional logistic regression analysis between the four protective HLA alleles in SSc.

*HLA* allele	Unconditioned		Conditioned on DRB1*13:*02*		Conditioned on DRB1*14:*06*		Conditioned on DQB1*03:*01*		Conditioned on DPB1*02:*01*	
	*P*	OR (95%CI)	*P*_adjusted_	OR_adjusted_ (95%CI)	*P*_adjusted_	OR_adjusted_ (95%CI)	*P*_adjusted_	OR_adjusted_ (95%CI)	*P*_adjusted_	OR_adjusted_ (95%CI)
DRB1*13:*02*	0.0006	0.48(0.31–0.73)	NA	NA	0.0005	0.47 (0.30–0.71)	0.0002	0.45 (0.29–0.69)	0.0006	0.47 (0.31–0.73)
DRB1*14:*06*	0.0046	0.05(0.01–0.41)	0.0040	0.05 (0.01–0.39)	NA	NA	0.0127	0.07 (0.01–0.57)	0.0046	0.05 (0.01–0.40)
DQB1*03:*01*	0.0013	0.59(0.43–0.82)	0.0005	0.56 (0.41–0.78)	0.0264	0.69 (0.50–0.96)	NA	NA	0.0019	0.60 (0.43–0.83)
DPB1*02:*01*	8.91X10^-6^	0.58(0.46–0.74)	8.71X10^-6^	0.58 (0.45–0.74)	9.87X10^-6^	0.58 (0.45–0.74)	1.22X10^-5^	0.58 (0.46–0.74)	NA	NA

SSc: systemic sclerosis, OR: odds ratio, CI: confidence interval, NA not applicable. *P*, OR, 95%CI, *P*_adjusted_, and OR_adjusted_ were calculated by logistic regression analysis under the additive model.

When haplotype frequencies were compared between SSc patients and controls, a tendency for four haplotypes (DRB1*15:01-DQB1*06:02-DPB1*02:*01*, DRB1*13:02-DQB1*06:04-DPB1*04:*01*, DRB1*04:06-DQB1*03:02-DPB1*02:*01*, DRB1*13:02-DQB1*06:04-DPB1*02:*01*) to be protectively associated was observed (S1 Table). These four protective haplotypes include the abovementioned independent protective alleles.

### *HLA* associations in SSc patients with ACA or ATA

We tested whether *HLA* class II alleles were associated with SSc with ACA. A significant association with susceptibility to SSc with ACA was found for the DRB1*01:*01* and DRB1*10:*01* alleles (*P* = 0.0001, *P*c = 0.0042, OR 2.52, 95%CI 1.58–4.01; *P* = 0.0003, *P*c = 0.0097, OR 11.17, 95%CI 2.42–51.48, respectively, S2 Table). A strong predisposing association between the carrier frequency of DQB1*05:*01* and SSc with ACA (*P* = 1.18X10^-6^, *P*c = 1.89X10^-5^, OR 3.07, 95%CI 1.97–4.80, S3 Table) was detected. On the other hand, DQB1*03:*01* was associated with resistance to SSc with ACA (*P* = 1.05X10^-5^, *Pc* = 0.0002, OR 0.32, 95%CI 0.18–0.55), despite the fact that this allele is known to be associated with susceptibility to SSc with ACA in Europeans [6]. DPB1*04:*02* was associated with SSc with ACA (*P* = 0.0001, *P*c = 0.0020, OR 2.23, 95%CI 1.48–3.35, S4 Table). A tendency for the four class II alleles, DRB1*13:*02*, DRB1*14:*06*, DQB1*03:*01*, and DPB1*02:*01*, to be protectively associated with SSc with ACA was also observed (S2, S3 and S4 Tables). The similar tendency for the ACA associated alleles to be associated with lcSSc was observed (S2, S3 and S4 Tables), though it was weaker.

We then compared the allele carrier frequencies of *DRB1*, *DQB1*, and *DPB1* in SSc with ATA with their frequencies in healthy controls. A significant association with susceptibility to SSc with ATA was found for the DRB1*15:*02* allele (*P* = 7.22X10^-9^, *P*c = 2.02X10^-7^, OR 3.58, 95%CI 2.33–5.50, S2 Table). A predisposing association between the carrier frequency of DQB1*06:*01* and SSc with ATA (*P* = 3.20X10^-5^, *P*c = 0.0005, OR 2.41, 95%CI 1.59–3.64, S3 Table) was detected. DQB1*06:*04* was associated with resistance to SSc with ATA (*P* = 2.95X10^-5^, *P*c = 0.0004, OR 0.06, 95%CI 0.01–0.45) and it was known that DQB1*06:*04* and protective DRB1*13:*02* are in strong linkage disequilibrium [26]. DPB1*03:*01* and DPB1*09:*01* are associated with SSc with ATA (*P* = 3.42X10^-5^, *P*c = 0.0006, OR 3.32, 95%CI 1.92–5.74; *P* = 7.82X10^-12^, *P*c = 1.33X10^-10^, OR 4.54, 95%CI 2.94–7.01, respectively, S4 Table). A protective association for DPB1*02:*01* and DPB1*04:*01* was detected with SSc with ATA (*P* = 2.06X10^-8^, *P*c = 3.50X10^-7^, OR 0.24, 95%CI 0.14–0.42; *P* = 4.18X10^-5^, *P*c = 0.0007, OR 0.04, 95%CI 0.00–0.62, respectively, S4 Table). A tendency for the four class II alleles, DRB1*13:*02*, DRB1*14:*06*, DQB1*03:*01*, and DPB1*02:*01*, to be protectively associated with SSc with ATA was observed (S2, S3 and S4 Tables). The similar tendency for the ATA associated alleles to be associated with dcSSc was observed (S2, S3 and S4 Tables). Thus, different predisposing associations of *DRB1*, *DQB1* or *DPB1* alleles were detected in SSc with ACA or ATA.

We tested whether *HLA* class II alleles were associated with ACA positive lcSSc, ACA negative lcSSc, ATA positive dcSSc, or ATA negative dcSSc (S5 Table). The predisposing or protective associations of *DRB1*, *DQB1* or *DPB1* alleles were mainly detected in ACA positive lcSSc or ATA positive dcSSc, suggesting that *HLA* class II alleles specifically influence the production of antibodies rather than the development of clinical subtypes of SSc.

### Certain amino acid residues in the HLA-DRβ, DQβ, and DPβ chains are associated with SSc, or SSc with ACA or ATA

**Fig 1:**
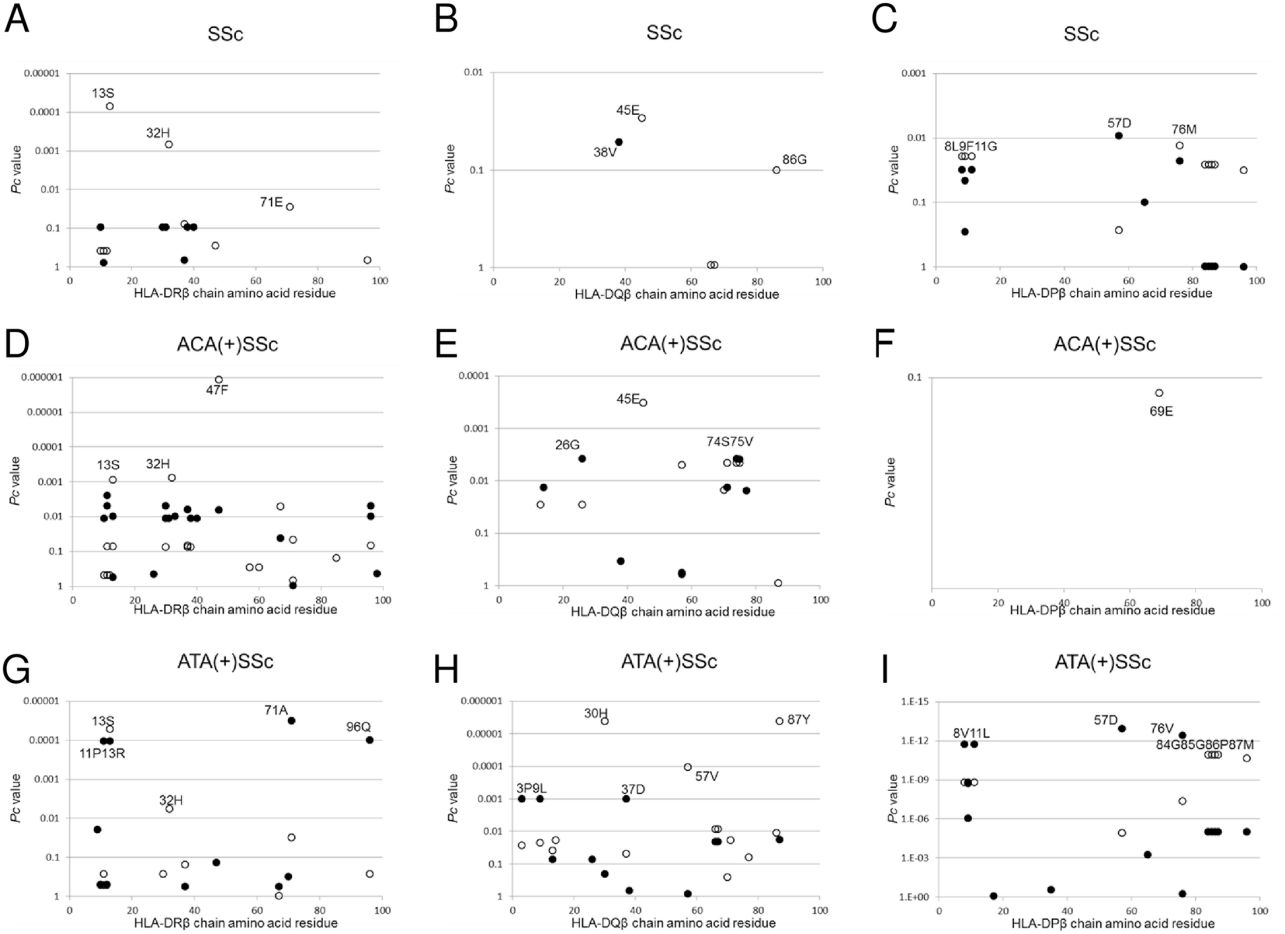
Associations of amino acid residues in the DRβ (A, D, G), DQβ (B, E, H), or DPβ (C, F, I) chain with SSc (A, B, C), anti-centromere antibody- positive [ACA(+)] SSc (D, E, F), and anti-topoisomerase I antibody-positive [ATA(+)] SSc (G, H, I). Corrected *P* (*P*c) values were calculated by multiplying the *P* value by the number of amino acid residues tested. Associations were established by Fisher’s exact test using 2X2 contingency tables. Predisposing associations are indicated by filled circles and protective associations by open circles.

Finally, we analyzed the association with SSc with respect to each amino acid residue in the HLA-DRβ, DQβ, and DPβ chains. Serine at position 13 (13S, *P* = 2.06X10^-6^, OR = 0.49, *P*c = 7.00X10^-5^, 95% CI 0.36–0.66) in the DRβ chain showed a strong protective association with SSc (Fig 1A, open circles). Glutamic acid at position 45 (45E, *P* = 0.0009, OR = 0.56, *P*c = 0.0291, 95% CI 0.40–0.79) in the DQβ chain showed a protective association with SSc (Fig 1B, open circle), whereas aspartic acid at position 57 (57D, *P* = 0.0005, OR = 1.64, *P*c = 0.0092, 95% CI 1.24–2.17) in the DPβ chain showed a predisposing association with SSc (Fig 1C, filled circle).

We also analyzed the association with SSc with ACA with respect to each amino acid residue in the HLA-DRβ, DQβ, and DPβ chains. Phenylalanine at position 47 (47F, *P* = 3.43X10^-8^, OR = 0.37, *P*c = 1.17X10^-6^, 95% CI 0.26–0.53) in the DRβ chain showed strong protective associations with SSc with ACA (Fig 1D, open circles). Glutamic acid at position 45 (45E, *P* = 1.05X10^-5^, OR = 0.32, *P*c = 0.0003, 95% CI 0.18–0.55) in the DQβ chain showed protective associations with SSc with ACA (Fig 1E, open circle). No statistically significant association was found for amino acid residue in the DPβ chain.

In addition, we analyzed the association with SSc with ATA with respect to each amino acid residue in the HLA-DRβ, DQβ, and DPβ chains. Serine at position 13 (13S, *P* = 1.52X10^-6^, OR = 0.29, *P*c = 5.16X10^-5^, 95% CI 0.17–0.50) in the DRβ chain showed a strong protective association with SSc with ATA (Fig 1G, open circle), whereas alanine at position 71 (71A, *P* = 9.37X10^-7^, OR = 2.83, *P*c = 3.19X10^-5^, 95% CI 1.86–4.31) in the DRβ chain showed a strong predisposing association with SSc with ATA (Fig 1G, closed circle). Histidine at position 30 (30H, *P* = 1.33X10^-7^, OR = 0.22, *P*c = 4.11X10^-6^, 95% CI 0.12–0.42), and tyrosine at position 87 (87Y, *P* = 1.32X10^-7^, OR = 0.22, *P*c = 4.11X10^-6^, 95% CI 0.12–0.42) in the DQβ chain showed a protective association with SSc with ACA (Fig 1H, open circle). Aspartic acid at position 57 (57D, *P* = 6.66X10^-15^, OR = 5.44, *P*c = 1.13X10^-13^, 95% CI 3.49–8.48), valine at position 76 (76V, P = 2.20X10^-14^, OR = 5.27, Pc = 3.74X10^-13^, 95% CI 3.39–8.18) in the DPβ chain showed a predisposing association with SSc (Fig 1I, filled circle). Thus, association analysis suggested roles for specific amino acid residues in the HLA-DRβ, DQβ, and DPβ chains.

## Discussion

Several studies have noted predisposing associations of *HLA* class II alleles with SSc [6,8,9,13,14]. However, few studies for the *HLA* protective association have been validated in SSc. DRB1*07:*01*, DRB1*15:*01*, DQB1*02:*02*, and DQB1*06:*02* were protectively associated with European SSc [6], and DRB1*01:*01*, DRB1*04:*06*, DRB1*07:*01*, and DPB1*02:*01* were with Chinese SSc [13,14]. The present study reports significant protective associations of the four alleles, DRB1*13:*02*, DRB1*14:*06*, DQB1*03:*01*, and DPB1*02:*01*, with Japanese SSc (Table 2), though these protective associations except for DPB1*02:*01* were not observed in previous studies. A lower frequency of DR6 alleles in Asian patients with SSc has been reported, so far [9,13]. The protective effect of DR6 seems to be partly mediated by DRB1*13:*02*, which is a common protective allele for several autoimmune diseases in Japanese [21,22,27]. It was known that DRB1*14:06 and DQB1*03:*01* are in strong linkage disequilibrium in the Japanese population [26]. However, conditional logistic regression analysis between them in SSc revealed that they independently affected on the disease protection (Table 4). Because of the limited sample size of this study, the observed protective association was modest. The protective association of the four *HLA* class II alleles with SSc should be confirmed in future large scale studies.

In this study, we found a protective association of DQB1*03:*01* with Japanese SSc. This protective effect was also observed for the SSc with ACA (S3 Table). However, our findings are not consistent with a previous report that DQB1*03:*01* is a risk allele for SSc with ACA in the European population [6]. This could be explained by the linkage disequilibrium of *DRB11*-DQB1*03:*01* in European populations [7]. However, we cannot rule out the possibility that there are other causative genes for SSc with ACA in the *HLA* region in linkage disequilibrium with the culprit gene in the DQB1*03:*01* haplotype. This possibility could be addressed by comparison of the re-sequencing data of the entire *HLA* region of DQB1*03:*01* haplotype in Japanese and Europeans.

The present study reports a significant predisposing association of DRB1*01:*01* and DRB1*10:*01*, DQB1*05:*01*, and DPB1*04:*02* with Japanese ACA positive SSc. The predisposing associations of DRB1*10:*01* and DRB1*15:*02* with ACA positive SSc in Chinese were reported [13]. On the other hand, DRB1*01:*01* and DQB1*05:*01* were associated with European ACA positive SSc [6]. The association of DPB1*04:*02* with Japanese ACA positive SSc was also reported [9]. Our findings are consistent with these previous reports. The higher haplotype frequency of DRB1*01:01-DQB1*05:*01* and DRB1*10:01-DQB1*05:*01* in the Japanese population suggests an important role of DQB1*05:*01* allele in the pathogenesis of ACA positive SSc in Japanese [26]. No associations with DQB1*26 epi including DQB1*03:*01* and DQB1*06:*01* were detected in the present study, suggesting that no DQB1*26 epi alleles other than DQB1*05:*01* are risk factors for ACA positive SSc in Japanese.

In the present study, the carrier frequencies of the DRB1*15:*02*, DQB1*06:*01*, DPB1*03:*01*, and DPB1*09:*01* alleles were higher in SSc patients with ATA. Associations between DRB1*15:*02*, DQB1*06:*01*, and DPB1*09:*01* and the presence of ATA have been reported in Japanese SSc patients [9]. The predisposing association of DRB1*15:*02*, DRB1*16:*02*, DPB1*03:*01* and DPB1*13:*01* with ATA positive SSc in Chinese was also reported [13,14]. DRB1*11:*04*, DQB1*03:*01*, and DPB1*13:*01* were strongly associated with ATA positive SSc in European descents [6]. The predisposing alleles in our study are overlapping with those reported in the previous reports.

We revealed that amino acid residues 13, 32 and 71 of the HLA-DRβ chain were protectively associated with SSc (Fig 1A). Amino acid residues 13, 32 and 71 form the HLA-DR peptide-binding groove [28], suggesting the involvement of peptide antigens bound to specific HLA-DR molecules in controlling the prevention of SSc. It was also found that other amino acid residues of the DRβ, DQβ, or DPβ chains were associated with SSc with ACA or ATA (Fig 1D–1I), though they are different from the results from the previous study in European populations [5]. These would be reflected by the ethnic differences of susceptible and protective *HLA* alleles [2,6,7]. This information suggests that peptide antigens loaded on specific HLA alleles controlled the generation of autoantibodies.

Because the distribution of *HLA* alleles in other ethnic populations is different from the Japanese, the role of some *HLA* class II alleles in SSc in other populations should be determined. Thus, the present study identified protective associations of *HLA* class II alleles with Japanese SSc; our findings support independent protective roles for the four class II alleles, DRB1*13:*02*, DRB1*14:*06*, DQB1*03:*01*, and DPB1*02:*01*, in the pathogenesis of SSc.

## Supporting Information

S1 Fig The statistical power in each condition of allele carrier frequency and odds ratio was calculated on the comparison between the overall SSc and the control. (PDF)

S1 Table HLA haplotype frequency in the SSc patients and controls. SSc: systemic sclerosis. Haplotypes with more than 1% frequency in controls are shown.
(PDF)

S2 Table HLA-DRB1 allele carrier frequencies in the SSc subsets and the control. SSc: systemic sclerosis, dcSSc: diffuse cutaneous SSc, lcSSc: limited cutaneous SSc, ACA: anti-centromere antibodies, ATA: anti-topoisomerase antibodies, OR: odds ratio, CI: confidence interval, Pc: corrected P value, NS: not significant. Allele carrier frequencies are shown in parenthesis (%). Association was tested between the SSc subsets and the control by Fisher's exact test using 2X2 contingency tables under the dominant model.
(PDF)

S3 Table HLA-DQB1 allele carrier frequencies in the SSc subsets and the control. SSc: systemic sclerosis, dcSSc: diffuse cutaneous SSc, lcSSc: limited cutaneous SSc, ACA: anti-centromere antibodies, ATA: anti-topoisomerase antibodies, OR: odds ratio, CI: confidence interval, Pc: corrected P value, NS: not significant. Allele carrier frequencies are shown in parenthesis (%). Association was tested between the SSc subsets and the control by Fisher's exact test using 2X2 contingency tables under the dominant model.
(PDF)

S4 Table HLA-DPB1 allele carrier frequencies in the SSc subsets and the control. SSc: systemic sclerosis, dcSSc: diffuse cutaneous SSc, lcSSc: limited cutaneous SSc, ACA: anti-centromere antibodies, ATA: anti-topoisomerase antibodies, OR: odds ratio, CI: confidence interval, Pc: corrected P value, NS: not significant. Allele carrier frequencies are shown in parenthesis (%). Association was tested between the SSc subsets and the control by Fisher's exact test using 2X2 contingency tables under the dominant model.
(PDF)

S5 Table HLA class II allele carrier frequencies in the SSc subsets and the control. SSc: systemic sclerosis, dcSSc: diffuse cutaneous SSc, lcSSc: limited cutaneous SSc, ACA: anti-centromere antibodies, ATA: anti-topoisomerase antibodies, OR: odds ratio, CI: confidence interval, Pc: corrected P value, NS: not significant. Allelecarrier frequencies are shown in parenthesis (%). Association was tested between the SSc subsets and the control byFisher's exact test using 2X2 contingency tables under the dominant model.
(PDF)
