# Supplementary material for: Human Leukocyte Antigen and Systemic Sclerosis in Japanese: The Sign of the Four Independent Protective Alleles, DRB1*13:02, DRB1*14:06, DQB1*03:01, and DPB1*02:01
Source: PLoS One. 2016 Apr 26;11(4):e0154255. doi: 10.1371/journal.pone.0154255 (PMC4846066; doi:10.1371/journal.pone.0154255)
Supplement: S1 Fig — (PDF) [file pone.0154255.s001.pdf]

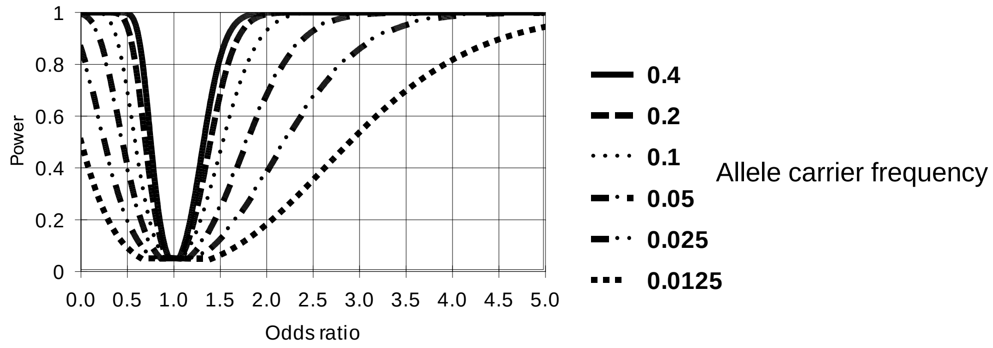


Supplementary Figure 1. The statistical power in each condition of allele carrier frequency and odds ratio was calculated on the comparison between the overall SSc and the control.
