# Supplementary material for: Human Leukocyte Antigen and Systemic Sclerosis in Japanese: The Sign of the Four Independent Protective Alleles, DRB1*13:02, DRB1*14:06, DQB1*03:01, and DPB1*02:01
Source: PLoS One. 2016 Apr 26;11(4):e0154255. doi: 10.1371/journal.pone.0154255 (PMC4846066; doi:10.1371/journal.pone.0154255)
Supplement: S1 Table — SSc: systemic sclerosis. Haplotypes with more than 1% frequency in controls are shown. (PDF) [file pone.0154255.s002.pdf]

Supplementary Table 1. *HLA* haplotype frequency in the SSc patients and controls.

DRB1	*DQB1*	*DPB1*	Case	Control	*P*
**15:01*	**06:02*	**02:01*	0.0189	0.0443	0.0025
**13:02*	**06:04*	**04:01*	0.0237	0.0460	0.0117
**04:06*	**03:02*	**02:01*	0.0083	0.0232	0.0189
**13:02*	**06:04*	**02:01*	0.0018	0.0115	0.0302
**15:02*	**06:01*	**09:01*	0.1251	0.0929	0.0783
**01:01*	**05:01*	**04:02*	0.0589	0.0396	0.0799
**04:05*	**04:01*	**04:02*	0.0050	0.0148	0.0854
**08:03*	**06:01*	**02:01*	0.0118	0.0206	0.1808
**08:02*	**03:02*	**02:01*	0.0053	0.0127	0.1928
**08:03*	**06:01*	**05:01*	0.0423	0.0307	0.2038
**15:01*	**06:02*	**05:01*	0.0192	0.0286	0.2117
**11:01*	**03:01*	**05:01*	0.0074	0.0125	0.3412
**08:02*	**03:02*	**05:01*	0.0198	0.0139	0.3516
**04:06*	**03:02*	**05:01*	0.0168	0.0123	0.4326
**09:01*	**03:03*	**05:01*	0.0858	0.0749	0.4801
**08:03*	**06:01*	**02:02*	0.0261	0.0210	0.5296
**14:54*	**05:03*	**05:01*	0.0090	0.0109	0.6389
**04:03*	**03:02*	**02:01*	0.0082	0.0113	0.6392
**09:01*	**03:03*	**02:01*	0.0330	0.0374	0.6984
**04:05*	**04:01*	**05:01*	0.0668	0.0674	1.0000
**04:05*	**04:01*	**02:01*	0.0135	0.0128	1.0000
**14:03*	**03:01*	**05:01*	0.0122	0.0121	1.0000

SSc: systemic sclerosis. Haplotypes with more than 1% frequency in controls are shown.
