# Supplementary material for: Human Leukocyte Antigen and Systemic Sclerosis in Japanese: The Sign of the Four Independent Protective Alleles, DRB1*13:02, DRB1*14:06, DQB1*03:01, and DPB1*02:01
Source: PLoS One. 2016 Apr 26;11(4):e0154255. doi: 10.1371/journal.pone.0154255 (PMC4846066; doi:10.1371/journal.pone.0154255)
Supplement: S2 Table — SSc: systemic sclerosis, dcSSc: diffuse cutaneous SSc, lcSSc: limited cutaneous SSc, ACA: anti-centromere antibodies, ATA: anti-topoisomerase antibodies, OR: odds ratio, CI: confidence interval, Pc: corrected P value, NS: not significant. Allele carrier frequencies are shown in parenthesis (%). Association was tested between the SSc subsets and the control by Fisher's exact test using 2X2 contingency tables under the dominant model. (PDF) [file pone.0154255.s003.pdf]

Supplementary Table 2. *HLA-DRB1* allele carrier frequencies in the SSc subsets and the control.

		dcSSc (n=157)	lcSSc (n=266)	ACA(+)SSc (n=194)	ATA(+)SSc (n=119)	Control (n=413)
*DRB1*01:01*	Numbe	11 (7.0)	52 (19.5)	43 (22.2)	3 (2.5)	42 (10.2)
	P	0.3322	0.0009	0.0001	0.0078	
	OR	0.67	2.15	2.52	0.23	
	*P*c	NS	0.0265	0.0042	0.2180	
	95%CI		(1.38–3.33)	(1.58–4.01)	(0.07–0.75)	
*DRB1*10:01*	Numbe	2 (1.3)	12 (4.5)	10 (5.2)	1 (0.8)	2 (0.5)
	P	0.3050	0.0004	0.0003	0.5329	
	OR	2.65	9.71	11.17	1.74	
	*P*c	NS	0.0129	0.0097	NS	
	95%CI		(2.16–43.73)	(2.42–51.48)		
*DRB1*13:02*	Numbe	9 (5.7)	21 (7.9)	14 (7.2)	3 (2.5)	57 (13.8)
	P	0.0078	0.0192	0.0208	0.0002	
	OR	0.38	0.54	0.49	0.16	
	*P*c	0.2185	0.5754	0.6252	0.0062	
	95%CI	(0.18–0.79)	(0.32–0.91)	(0.26–0.90)	(0.05–0.53)	
*DRB1*14:06*	Numbe	0 (0.0)	1 (0.4)	1 (0.5)	0 (0.0)	16 (3.9)
	P	0.0085	0.0041	0.0170	0.0290	
	OR	0.08	0.09	0.13	0.10	
	*P*c	0.2381	0.1233	0.5102	0.8122	
	95%CI	(0.00–1.28)	(0.01–0.71)	(0.02–0.98)	(0.01–1.69)	
*DRB1*15:02*	Numbe	59 (37.6)	50 (18.8)	27 (13.9)	59 (49.6)	89 (21.5)
	P	0.0002	0.4359	0.0269	7.22×10^{-9}	
	OR	2.19	0.84	0.59	3.58	
	*P*c	0.0047	NS	0.8077	2.02×10^{-7}	
	95%CI	(1.47–3.27)		(0.37–0.94)	(2.33–5.50)	
DR6 (*DRB1*13, *14*)	Numbe	27 (17.2)	51 (19.2)	36 (18.6)	12 (10.1)	137 (33.2)
	P	0.0001	7.24×10^{-5}	0.0002	2.17×10^{-7}	
	OR	0.42	0.48	0.46	0.23	
	95%CI	(0.26–0.66)	(0.33–0.69)	(0.30–0.70)	(0.12–0.42)	

SSc: systemic sclerosis, dcSSc: diffuse cutaneous SSc, lcSSc: limited cutaneous SSc, ACA: anti-centromere antibodies, ATA: anti-topoisomerase I antibodies, ILD: interstitial lung disease, PAH: pulmonary arterial hypertension, OR: odds ratio, CI: confidence interval, *P*c: corrected *P* value, NS: not significant. Allele carrier frequencies are shown in parenthesis (%). Association was tested between the SSc subsets and the control by
