# Supplementary material for: Human Leukocyte Antigen and Systemic Sclerosis in Japanese: The Sign of the Four Independent Protective Alleles, DRB1*13:02, DRB1*14:06, DQB1*03:01, and DPB1*02:01
Source: PLoS One. 2016 Apr 26;11(4):e0154255. doi: 10.1371/journal.pone.0154255 (PMC4846066; doi:10.1371/journal.pone.0154255)
Supplement: S3 Table — SSc: systemic sclerosis, dcSSc: diffuse cutaneous SSc, lcSSc: limited cutaneous SSc, ACA: anti-centromere antibodies, ATA: anti-topoisomerase antibodies, OR: odds ratio, CI: confidence interval, Pc: corrected P value, NS: not significant. Allele carrier frequencies are shown in parenthesis (%). Association was tested between the SSc subsets and the control by Fisher's exact test using 2X2 contingency tables under the dominant model. (PDF) [file pone.0154255.s004.pdf]

Supplementary Table 3. *HLA-DQB1* allele carrier frequencies in the SSc subsets and the control.

		dcSSc (n=157)	lcSSc (n=266)	ACA(+)SSc (n=194)	ATA(+)SSc (n=119)	Control (n=413)
*DQB1*03:01*	Number	30 (19.1)	32 (12.0)	17 (8.8)	24 (20.2)	96 (23.2)
	P	0.3109	0.0003	1.05×10^{-5}	0.5348	
	OR	0.78	0.45	0.32	0.83	
	*P*c	NS	0.0044	0.0002	NS	
	95%CI		(0.29-0.70)	(0.18-0.55)		
*DQB1*05:01*	Number	13 (8.3)	63 (23.7)	52 (26.8)	4 (3.4)	44 (10.7)
	P	0.4388	8.10×10^{-6}	1.18×10^{-6}	0.0112	
	OR	0.76	2.60	3.07	0.29	
	*P*c	NS	0.0001	1.89×10^{-5}	0.1673	
	95%CI		(1.71-3.97)	(1.97-4.80)	(0.10-0.83)	
*DQB1*06:01*	Number	77 (49.0)	89 (33.5)	60 (30.9)	67 (56.3)	144 (34.9)
	P	0.0021	0.7408	0.3578	3.20×10^{-5}	
	OR	1.80	0.94	0.84	2.41	
	*P*c	0.0316	NS	NS	0.0005	
	95%CI	(1.24-2.61)			(1.59-3.64)	
*DQB1*06:04*	Number	8 (5.1)	21 (7.9)	15 (7.7)	1 (0.8)	50 (12.1)
	P	0.0128	0.0947	0.1217	2.95×10^{-5}	
	OR	0.39	0.62	0.61	0.06	
	*P*c	0.1914	NS	NS	0.0004	
	95%CI	(0.18-0.84)			(0.01-0.45)	

SSc: systemic sclerosis, dcSSc: diffuse cutaneous SSc, lcSSc: limited cutaneous SSc, ACA: anti-centromere antibodies, ATA: anti-topoisomerase I antibodies, ILD: interstitial lung disease, PAH: pulmonary arterial hypertension, OR: odds ratio, CI: confidence interval, *P*c: corrected *P* value, NS: not significant. Allele carrier frequencies are shown in parenthesis (%). Association was tested between the SSc subsets and the control
