# Supplementary material for: Human Leukocyte Antigen and Systemic Sclerosis in Japanese: The Sign of the Four Independent Protective Alleles, DRB1*13:02, DRB1*14:06, DQB1*03:01, and DPB1*02:01
Source: PLoS One. 2016 Apr 26;11(4):e0154255. doi: 10.1371/journal.pone.0154255 (PMC4846066; doi:10.1371/journal.pone.0154255)
Supplement: S4 Table — SSc: systemic sclerosis, dcSSc: diffuse cutaneous SSc, lcSSc: limited cutaneous SSc, ACA: anti-centromere antibodies, ATA: anti-topoisomerase antibodies, OR: odds ratio, CI: confidence interval, Pc: corrected P value, NS: not significant. Allele carrier frequencies are shown in parenthesis (%). Association was tested between the SSc subsets and the control by Fisher's exact test using 2X2 contingency tables under the dominant model. (PDF) [file pone.0154255.s005.pdf]

Supplementary Table 4. *HLA-DPB1* allele carrier frequencies in the SSc subsets and the control.

		dcSSc (n=157)	lcSSc (n=266)	ACA(+)SSc (n=194)	ATA(+)SSc (n=119)	Control (n=413)
*DPB1*02:01*	Numbe	30 (19.1)	85 (32.0)	64 (33.0)	18 (15.1)	175 (42.4)
	P	$1.13X10^{-7}$	0.0076	0.0324	$2.06X10^{-8}$	
	OR	0.32	0.64	0.67	0.24	
	*P*c	$1.81X10^{-6}$	0.1210	0.4860	$3.50X10^{-7}$	
	95%CI	(0.21–0.50)	(0.46–0.88)	(0.47–0.96)	(0.14–0.42)	
*DPB1*03:01*	Numbe	20 (12.7)	41 (15.4)	21 (10.8)	28 (23.5)	35 (8.5)
	P	0.1520	0.0060	0.3685	$3.42X10^{-5}$	
	OR	1.58	1.97	1.31	3.32	
	*P*c	NS	0.0965	NS	0.0006	
	95%CI		(1.22–3.18)		(1.92–5.74)	
*DPB1*04:01*	Numbe	10 (6.4)	20 (7.5)	17 (8.8)	0 (0.0)	41 (9.9)
	P	0.2494	0.3364	0.7674	$4.18X10^{-5}$	
	OR	0.62	0.74	0.87	0.04	
	*P*c	NS	NS	NS	0.0007	
	95%CI				(0.00–0.62)	
*DPB1*04:02*	Numbe	21 (13.4)	68 (25.6)	57 (29.4)	7 (5.9)	65 (15.7)
	P	0.5154	0.0021	0.0001	0.0057	
	OR	0.83	1.84	2.23	0.33	
	*P*c	NS	0.0333	0.0020	0.0963	
	95%CI		(1.25–2.69)	(1.48–3.35)	(0.15–0.75)	
*DPB1*09:01*	Numbe	62 (39.5)	50 (18.8)	28 (14.4)	63 (52.9)	82 (19.9)
	P	$2.93X10^{-6}$	0.7663	0.1145	$7.82X10^{-12}$	
	OR	2.63	0.93	0.68	4.54	
	*P*c	$4.69X10^{-5}$	NS	NS	$1.33X10^{-10}$	
	95%CI	(1.76–3.93)			(2.94–7.01)	

SSc: systemic sclerosis, dcSSc: diffuse cutaneous SSc, lcSSc: limited cutaneous SSc, ACA: anti-centromere antibodies, ATA: anti-topoisomerase I antibodies, ILD: interstitial lung disease, PAH: pulmonary arterial hypertension, OR: odds ratio, CI: confidence interval, *P*c: corrected *P* value, NS: not significant. Allele carrier frequencies are shown in parenthesis (%). Association was tested between the SSc subsets
