# Supplementary material for: Human Leukocyte Antigen and Systemic Sclerosis in Japanese: The Sign of the Four Independent Protective Alleles, DRB1*13:02, DRB1*14:06, DQB1*03:01, and DPB1*02:01
Source: PLoS One. 2016 Apr 26;11(4):e0154255. doi: 10.1371/journal.pone.0154255 (PMC4846066; doi:10.1371/journal.pone.0154255)
Supplement: S5 Table — SSc: systemic sclerosis, dcSSc: diffuse cutaneous SSc, lcSSc: limited cutaneous SSc, ACA: anti-centromere antibodies, ATA: anti-topoisomerase antibodies, OR: odds ratio, CI: confidence interval, Pc: corrected P value, NS: not significant. Allelecarrier frequencies are shown in parenthesis (%). Association was tested between the SSc subsets and the control byFisher's exact test using 2X2 contingency tables under the dominant model. (PDF) [file pone.0154255.s006.pdf]

Supplementary Table 5. *HLA* class II allele carrier frequencies in the SSc subsets and the control.

		ATA(+)dcSSc (n=85)	ATA(−)dcSSc (n=71)	ACA(+)lcSSc (n=167)	ACA(−)lcSSc (n=90)	Control (n=413)
*DRB1*01:01*	Number	1 (1.2)	10 (14.1)	40 (24.0)	7 (7.8)	42 (10.2)
	P	0.0047	0.3053	$3.61X10^{-5}$	0.5618	
	OR	0.11	1.45	2.78	0.74	
	*P*c	0.1323	NS	0.0011	NS	
	95%CI	(0.01−0.77)		(1.73−4.49)		
*DRB1*10:01*	Number	0 (0.0)	2 (2.8)	9 (5.4)	3 (3.3)	2 (0.5)
	P	1.0000	0.1045	0.0004	0.0422	
	OR	0.96	5.96	11.71	7.09	
	*P*c	NS	NS	0.0108	NS	
	95%CI			(2.50−54.77)	(1.17−43.05)	
*DRB1*13:02*	Number	2 (2.4)	7 (9.9)	14 (8.4)	7 (7.8)	57 (13.8)
	P	0.0014	0.4503	0.0923	0.1612	
	OR	0.15	0.68	0.57	0.53	
	*P*c	0.0398	NS	NS	NS	
	95%CI	(0.04−0.63)				
*DRB1*14:06*	Number	0 (0.0)	0 (0.0)	1 (0.6)	0 (0.0)	16 (3.9)
	P	0.0869	0.1454	0.0314	0.0890	
	OR	0.14	0.17	0.15	0.13	
	*P*c	NS	NS	0.9426	NS	
	95%CI			(0.02−1.14)		
*DRB1*15:02*	Number	42 (49.4)	17 (23.9)	22 (13.2)	27 (30.0)	89 (21.5)
	P	$5.78X10^{-7}$	0.6432	0.0200	0.0974	
	OR	3.56	1.15	0.55	1.56	
	*P*c	$1.62X10^{-5}$	NS	0.5988	NS	
	95%CI	(2.19−5.78)		(0.33−0.92)		
DR6	Number	9 (10.6)	18 (25.4)	32 (19.2)	19 (21.1)	137 (33.2)
(*DRB1*13, *14*)	*P*	$1.19X10^{-5}$	0.2167	0.0008	0.0320	
	OR	0.24	0.68	0.48	0.54	
	95%CI	(0.12−0.49)		(0.31−0.74)	(0.31−0.93)	
*DQB1*03:01*	Number	18 (21.2)	12 (16.9)	13 (7.8)	16 (17.8)	96 (23.2)
	P	0.7772	0.2811	$6.43X10^{-6}$	0.3275	
	OR	0.89	0.67	0.28	0.71	
	*P*c	NS	NS	0.0001	NS	
	95%CI			(0.15−0.51)		
*DQB1*05:01*	Number	1 (1.2)	12 (16.9)	48 (28.7)	10 (11.1)	44 (10.7)
	P	0.0029	0.1572	$2.82X10^{-7}$	0.8525	
	OR	0.10	1.71	3.38	1.05	
	*P*c	0.0439	NS	$4.51X10^{-6}$	NS	
	95%CI	(0.01−0.73)		(2.14−5.35)		
*DQB1*06:01*	Number	48 (56.5)	29 (40.8)	49 (29.3)	38 (42.2)	144 (34.9)
	P	0.0003	0.3497	0.2076	0.2259	
	OR	2.42	1.29	0.78	1.37	
	*P*c	0.0051	NS	NS	NS	
	95%CI	(1.51−3.89)				
*DQB1*06:04*	Number	1 (1.2)	7 (9.9)	15 (9.0)	6 (6.7)	50 (12.1)
	P	0.0012	0.6932	0.3117	0.1937	
	OR	0.09	0.79	0.72	0.52	
	*P*c	0.0181	NS	NS	NS	
	95%CI	(0.01−0.63)				
*DPB1*02:01*	Number	10 (11.8)	20 (28.2)	57 (34.1)	27 (30.0)	175 (42.4)
	P	$2.77X10^{-8}$	0.0261	0.0754	0.0328	
	OR	0.18	0.53	0.70	0.58	
	*P*c	$4.43X10^{-7}$	0.3908	NS	0.5246	
	95%CI	(0.09−0.36)	(0.31−0.93)		(0.36−0.95)	
*DPB1*03:01*	Number	18 (21.2)	2 (2.8)	21 (12.6)	19 (21.1)	35 (8.5)
	P	0.0015	0.1431	0.1614	0.0011	
	OR	2.90	0.31	1.55	2.89	
	*P*c	0.0245	NS	NS	0.0182	
	95%CI	(1.55−5.42)			(1.57−5.34)	
*DPB1*04:01*	Number	0 (0.0)	10 (14.1)	16 (9.6)	4 (4.4)	41 (9.9)
	P	0.0007	0.2966	1.0000	0.1067	
	OR	0.05	1.49	0.96	0.42	
	*P*c	0.0113	NS	NS	NS	
	95%CI	(0.00−0.86)				
*DPB1*04:02*	Number	3 (3.5)	18 (25.4)	49 (29.3)	14 (15.6)	65 (15.7)
	P	0.0015	0.0597	0.0003	1.0000	
	OR	0.20	1.82	2.22	0.99	
	*P*c	0.0246	0.8962	0.0048	NS	
	95%CI	(0.06−0.64)		(1.45−3.40)		
*DPB1*09:01*	Number	45 (52.9)	17 (23.9)	22 (13.2)	27 (30.0)	82 (19.9)
	P	$2.30X10^{-9}$	0.4283	0.0724	0.0472	
	OR	4.54	1.27	0.61	1.73	
	*P*c	$3.67X10^{-8}$	NS	NS	0.7548	
	95%CI	(2.78−7.41)			(1.04−2.89)	

SSc: systemic sclerosis, dcSSc: diffuse cutaneous SSc, lcSSc: limited cutaneous SSc, ACA: anti−centromere antibodies, ATA: anti−topoisomerase antibodies, OR: odds ratio, CI: confidence interval, *P*c: corrected *P* value, NS: not significant. Allele carrier frequencies are shown in parenthesis (%). Association was tested between the SSc subsets and the control by Fisher's exact test using 2X2 contingency tables under the dominant model.
